# Identification of a hypervirulent extensively drug-resistant ST383 *Klebsiella pneumoniae* sub-lineage carrying ICEKp5 in Lebanon

**DOI:** 10.1128/aac.00384-26

**Published:** 2026-08-05

**Authors:** Martine Abboud, Tia Clara Chaaya, Yara Daccache, Nour El Alam, Theresa Gerges, Lea Haddad, Leon Kassabian, Jame Tannous, Yara Ghanem, Judy Nabbout, Kevin Chaar, Toni Nmeir, Anthony Haddad, Charbel Al Khoury, George F. Araj, Sima Tokajian

**Affiliations:** 1 Biological Sciences, Lebanese American University 37607, Byblos, Lebanon; 2 Biological Sciences, Lebanese American University, Beirut, Lebanon; 3 Department of Pathology and Laboratory Medicine, Faculty of Medicine, American University of Beirut Medical Center 66984, Beirut, Lebanon; Columbia University Irving Medical Center, New York, New York, USA

**Keywords:** CR-hvKp, OXA-48, NDM-5, ICEKp5, ST383, *Klebsiella pneumoniae*

## Abstract

*Klebsiella pneumoniae* ST383 has emerged as a high-risk clone, characterized by carbapenem resistance and increasing detection of hypervirulence determinants. We describe a phylogenetically distinct ST383 sub-lineage in Lebanon, defined by the acquisition of ICEKp5, which carries the yersiniabactin locus. Three ST383 *K. pneumoniae* clinical isolates (LBN_CAKp91, LBN_CTKp3, and LBN_CTKp11) recovered from a Lebanese medical center were subjected to whole-genome sequencing. Comparative genomic analysis included regional ST383 strains and previously characterized Lebanese isolates. The study isolates formed a tight, monophyletic cluster (three to nine single-nucleotide polymorphisms [SNPs]) that is phylogenetically distinct from the previously reported Lebanese ST383 clone (>164 SNPs) and grouped most closely to an Egyptian ST383 strain (59–65 SNPs). All three isolates carried ICEKp5 with yersiniabactin lineage *ybt14*, a feature absent in the earlier Lebanese ST383 clone. The isolates were the only ST383 strains to harbor the full spectrum of hypervirulence determinants to date, including capsule regulators (*rmpA* and *rmpA2*), aerobactin (*iucABCD* and *iutA*), yersiniabactin, and the hypervirulence biomarker *peg-344*. All isolates carried dual carbapenemases (*bla*_OXA-48_ and *bla*_NDM-5_) in addition to *bla*_CTX-M-15_ and *bla*_CTX-M-14b_. The genetic environments of *bla*_OXA-48_ and *bla*_NDM-5_ were highly conserved across geographically diverse ST383 isolates, indicating common plasmid origins. This study describes a phylogenetically distinct hypervirulent extensively drug-resistant (XDR) ST383 *K. pneumoniae* sub-lineage in Lebanon. The acquisition of ICEKp5, combined with plasmid-borne hypervirulence and resistance determinants, reveals the concerning convergence of hypervirulence and XDR. Enhanced surveillance and infection control measures are urgently needed to monitor this emerging high-risk clone.

## INTRODUCTION

A major public health threat has emerged through the convergence of hypervirulence and carbapenem resistance in *Klebsiella pneumoniae*, giving rise to carbapenem-resistant hypervirulent *K. pneumoniae* (CR-hvKp) strains (1). Convergence occurs either when CRKp strains acquire hypervirulence loci, including potent siderophore systems, and regulators of the mucoid phenotype (*rmpA* and *rmpA2*), or when hvKp acquire carbapenemase genes, frequently through large hybrid plasmids that form through recombination and fusion of distinct backbones (e.g., IncHI1B and IncFIB), enabling simultaneous multidrug resistance and heightened virulence (2, 3).

Multiple high-risk *K. pneumoniae* sequence types (STs) have emerged globally, including ST383 (Mediterranean/African) (4–7), ST11 (Asia), ST23 (worldwide), ST101 (Europe-Asia), ST258 (Americas/Europe) (8), and ST147, ST307, ST15 (9, 10). These clones exhibit distinct geographic distributions, resistance mechanisms, and virulence profiles.

ST383 has emerged as a prominent lineage across Lebanon, Egypt, Greece, and Qatar, although with variable prevalence. In Egypt, ST383 represented 41% of colistin-resistant ICU isolates in one cohort (11) and 14.7% of carbapenem-resistant bloodstream isolates in another (12). In Qatar, ST383 was less frequent overall (13), but most affected patients had recently traveled to Egypt, suggesting cross-border dissemination within the Eastern Mediterranean region (EMR). In Greece, among 378 KPC-2-producing *K. pneumoniae* isolates recovered during the 2009–2010 nationwide epidemic, nine belonged to ST383 and co-harbored *bla*_KPC-2_ and *bla*_VIM-4_ (7).

In Lebanon, ST383 accounted for 76% of carbapenem- and ceftazidime-avibactam-resistant isolates at a tertiary care center (6). Among these, five ST383 isolates carried NDM-5 and OXA-48, highlighting the establishment of this clone in the country. However, the hypervirulence potential of Lebanese ST383 strains has not been fully characterized.

This study reports the identification of a phylogenetically distinct ST383 *K. pneumoniae* sub-lineage in Lebanon, characterized by ICEKp5, which carries the yersiniabactin virulence locus. Through comprehensive genomic analysis, we aimed to characterize the virulence and resistance profiles of three clinical isolates, define their phylogenetic relationships with regional ST383 strains, and compare their genetic features with those of the previously reported Lebanese ST383 clone, which lacked ICEKp5 and its associated hypervirulence determinants.

## MATERIALS AND METHODS

### Bacterial strains collection and identification

Three non-duplicate clinical *K. pneumoniae* isolates were collected from the Clinical Microbiology Laboratory at the American University of Beirut Medical Center in Lebanon. Two isolates, designated LBN_CTKp3 and LBN_CTKp11, were collected in January 2025, whereas the third, designated as LBN_CAKp91, was collected in May 2025. The isolates were identified using matrix-assisted laser desorption ionization–time-of-flight mass spectrometry (MALDI-TOF MS) with the MALDI Biotyper software (Bruker Daltonics, Bremen, Germany). Carbapenem resistance was determined using the E-test method (AB BIODISK, Solna, Sweden) by testing the minimal inhibitory concentrations (MICs) of ertapenem, imipenem, and meropenem, with the results interpreted according to the Clinical Laboratory Standards Institute guidelines (14). The phenotypic detection of carbapenemase types was performed using the NG-Test CARBA-5 (Biotech SAS, Guipry, France), following the manufacturer’s instructions. For further analysis, the isolates were cultured on tryptic soy agar and then preserved in 50% glycerol at −20°C and −80°C.

### Antimicrobial susceptibility testing

The susceptibility of each isolate was determined using the Kirby-Bauer disk diffusion method. A saline suspension of each culture was plated on Mueller-Hinton agar. The following antibiotic disks were positioned over the inoculum: ciprofloxacin (5 µg), levofloxacin (5 µg), ofloxacin (5 µg), norfloxacin (10 µg), trimethoprim-sulfamethoxazole (1.25/23.75 µg), fosfomycin (200 µg), nitrofurantoin (100 µg), amikacin (30 µg), amoxicillin-clavulanic acid (20/10 µg), piperacillin-tazobactam (100/10 µg), ertapenem (10 µg), imipenem (10 µg), meropenem (10 µg), cefepime (30 µg), cefotaxime (30 µg), cefixime (5 µg), tetracycline (30 µg), tigecycline (15 µg), gentamicin (10 µg), colistin (10 µg), cefoxitin (30 µg), cefamandole (30 µg), cefuroxime (30 µg), and aztreonam (30 µg) (Bio-Rad, Hercules, CA, USA). After incubation at 37°C for 24 h, the results were interpreted according to the CLSI (14). Isolates were classified as multidrug-resistant (MDR) if resistant to at least one antimicrobial agent in three or more classes, extensively drug-resistant (XDR) if resistant to at least one agent in all but two or fewer categories, and pan-drug resistant (PDR) if non-susceptible to all agents in all antimicrobial categories (15).

### DNA extraction and whole-genome sequencing

Genomic DNA was extracted from overnight cultures of the isolates using the Sigma-Aldrich DNA extraction kit (Sigma-Aldrich; St. Louis, USA) following the manufacturer’s instructions. Genomic libraries were constructed using the Nextera XT DNA Library Preparation Kit (Illumina). Libraries were sequenced on an Illumina MiSeq using a paired-end 500-cycle protocol with a read length of 2 × 250 bp.

Long-read sequencing was performed using the Oxford Nanopore Technologies (ONT) platform. Sequencing libraries were prepared using the ONT Rapid Barcoding Kit V14 (SQK-RBK114.24/96), according to the manufacturer’s instructions. End-prep and native barcode ligation were performed using a 100–200 fmol sample diluted in 65 µL of nuclease-free water according to the Native Barcoding Kit amplicon protocol. Adapter ligation and cleaning were performed using NEB ligation and Agencourt AMPure XP beads (Beckman Coulter, USA), with the final adapter-ligated DNA library being 50–100 fmol. The library was loaded into an R10.4.1 flow cell (ONT) and sequenced using the MinION (ONT).

### PCR-based replicon typing

Characterization of plasmid incompatibility (Inc) groups was performed using the PBRT 2.0 kit (DIATHEVA, Fano, Italy) according to the manufacturer’s instructions to amplify 30 replicons found among *Enterobacterales*. The resulting products were visualized on a 2.5% agarose gel stained with ethidium bromide.

### Genome assembly, annotation, and analysis

FastQC v0.11.8 was used to assess the quality of Illumina raw reads (16). Trimmomatic v0.39 was used for adapter removal and trimming (17).

Nanopore raw data (fast5) were analyzed using Guppy Version 4.5.2 software (ONT) with a q-score threshold of 9. Basecalling was repeated using the following flags: –config dna_r9.4.1_450bps_hac. cfg–num_callers 4 –cpu_threads_per_caller 4. Barcodes were identified using –barcode_kits “EXP-NBD104 EXP-NBD114” and trimmed using –config configuration.cfg-trim_barcodes flags. Hybracter v0.12.0 was used for hybrid assembly, and the completeness and quality of the genomes were assessed using QUAST v5.3.0 (18, 19).

The genomes were annotated using the Bakta v1.11.4 pipeline with the v6.0 light database (20). Kleborate v3.2.4 was used for species identification, multilocus sequence typing, detection of virulence markers, and identification of integrative and conjugative elements (ICE) of *K. pneumoniae* (21). Virulence-associated genes were additionally cross-referenced using ABRicate v1.2.0 (22) against the Virulence Factor Database (VFDB) (https://www.mgc.ac.cn/VFs/). The capsule and lipopolysaccharide types were determined using Kaptive v3 (23). Antimicrobial resistance genes and chromosomal mutations were identified using Kleborate (21). Furthermore, acquired antibiotic, biocide, and heavy metal resistance genes were identified using AMRFinderPlus v4.2.5 with the “plus” database v2025-12-03.1 (24). Abricate v1.2.0 was used to detect fitness-associated genes (22).

Plasmid replicons were identified using PlasmidFinder v2.1.6 and further validated using the MOB-typer module of MOB-suite v3.1.9 (25).

### Comparative genomic analyses

Twenty-two high-quality ST383 *K. pneumoniae* genomes were retrieved from the NCBI database for comparative genomic analyses with our three study isolates, including comparisons of resistomes, virulomes, plasmid content, and genetic environments. The genomes were selected based on the assembly completeness and geographical distribution, with particular emphasis on regions with reported ST383 prevalence, including England, Qatar, and Italy. In addition, Lebanese ST383 isolates from a previously published study were also selected to ensure the integration of well-characterized regional strains into the data set (6). The accession numbers for these genomes are listed in Supplemental material 1.

### Phylogenetic tree construction and visualization

An SNP-based phylogenetic tree was constructed from 95 complete ST383 genomes retrieved from the NCBI, including the three study isolates (Supplemental material 1), with CHI_KP2010 used as an outgroup to root the tree. Then, the seven genomes showing the closest phylogenetic relatedness to the three study isolates, based on SNP distances, were selected, and an SNP distance matrix was generated for the resulting set of ten genomes (Supplemental material 2).

Core SNPs were called with Snippy v4.6 by aligning reads from each genome to QAT_10MC160002271, chosen as the reference for its high assembly quality and to maximize the shared core genome across closely related ST383 isolates (https://github.com/tseemann/snippy, accessed on 25 January 2021). High-quality SNPs and indels were annotated using SnpEff v5.1 after removal of low-confidence alleles with a consensus base quality <20 and a read depth <5 or a heterozygous base call (26). The parsimony tree was constructed from consensus core genome sequences generated by Snippy and kSNP3 v3.0 (27). The final output was visualized using iTOL (28).

### Graphical representation and visualization

The hypervirulence gene heatmap (Fig. 2b) was generated using Python v3.12 with the matplotlib and pandas libraries. Gene presence values were derived from Kleborate v3.2.4 output and cross-referenced with ABRicate v1.2.0 against the VFDB and AMRFinderPlus v4.2.5. All other graphs were generated in R v.3.6.1 using ggplot2, ggrepel, pheatmap, and ComplexHeatmap. Pairwise genomic alignments were visualized using clinker, with gene links displayed for pairwise amino acid identities exceeding 65%.

## RESULTS

### Comparative analysis of antibiotic resistance genes and plasmid replicons

**Fig 1:**
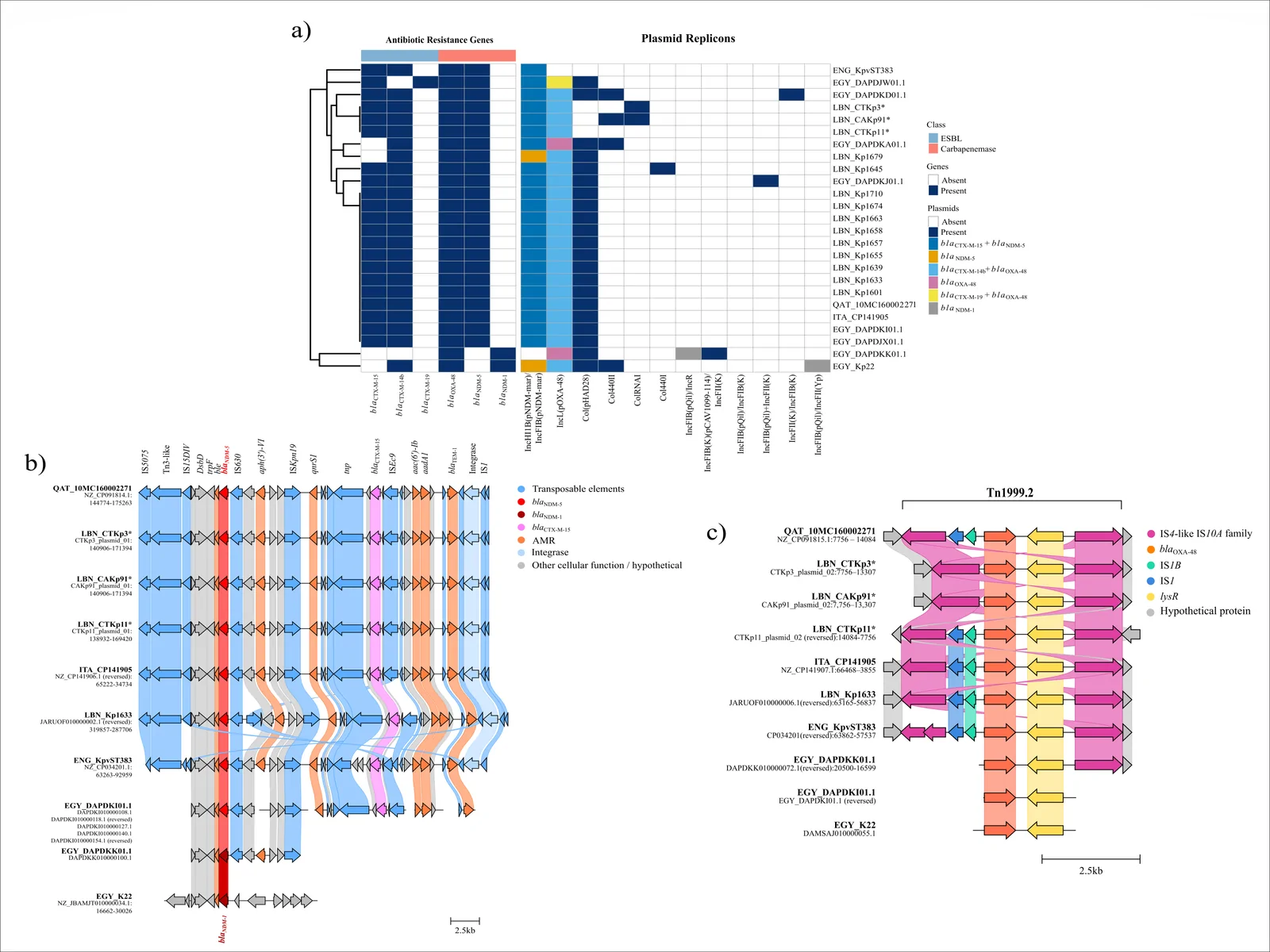
Distribution of antimicrobial resistance genes, plasmid replicons, and carbapenemase genetic contexts in ST383 *Klebsiella pneumoniae* isolates. (**a**) Heatmap of antibiotic resistance genes and plasmid replicons in ST383 *K. pneumoniae* isolates. Heatmap displaying the presence of antibiotic resistance genes (left panel) and plasmid replicons (right panel) across ST383 *K. pneumoniae* isolates. Resistance genes are grouped by class (color-coded bar at top): ESBL genes (*bla*_CTX-M-15_, *bla*_CTX-M-14b_, and *bla*_CTX-M-19_) and carbapenemase genes (*bla*_OXA-48_, *bla*_NDM-5_, and *bla*_NDM-1_). Dark blue indicates gene/plasmid presence, while white indicates absence. Plasmid replicons are color-coded to indicate associated resistance genes. Isolates are hierarchically clustered based on resistance and plasmid profiles (dendrogram, left). Isolates from the current study are marked with asterisks (*). The heatmap was generated using R v. 3.6.1. (**b**) Comparative analysis of the *bla*_NDM_ genetic environment in ST383 *K. pneumoniae* isolates. Linear comparison of the genetic regions flanking *bla*_NDM_ across ST383 *K. pneumoniae* isolates from different geographic origins. Genes are represented as arrows indicating transcriptional direction and are color-coded: *bla*_NDM_, transposases and insertion sequences (*tnp*, IS*15DIV*, IS*630*, IS*Kpn19*, and IS*Ec9*), bleomycin resistance (*ble*), aminoglycoside resistance genes (*aph(3')-VI*, *aac(6')-Ib*, and *aadA1*), quinolone resistance (*qnrS1*), and *bla*_TEM-1._ Colored ribbons connect homologous regions between isolates, illustrating the conserved synteny. The scale bar represents 2.5 kb. Isolate names and contig coordinates are shown on the left; the study isolate is marked with an asterisk (*). Structural variations in some isolates reflect fragmentation of draft genome assemblies across multiple contigs. (**c**) Comparative analysis of the *bla*_OXA-48_ genetic environment in ST383 *K. pneumoniae* isolates. Linear representation of the genetic regions flanking the *bla*_OXA-48_ across ST383 *K. pneumoniae* isolates from different geographic origins. Genes are represented as arrows indicating transcriptional direction and are color-coded: the *bla*_OXA-48_ (orange), insertion sequences and transposases (IS*10A*: pink; IS*1*: blue; IS*1B*: green), hypothetical proteins (gray), and *lysR* (yellow). Isolate names and contig coordinates are shown on the left. All isolates share a conserved genetic environment consisting of IS*10A-bla*_OXA-48_-*lysR*-IS*10A*. Incomplete structures in some isolates (EGY_DAPDKK01.1, EGY_DAPDKI01.1, and EGY_K22) reflect the fragmented nature of draft genome assemblies across multiple contigs rather than true genetic variation.

The three isolates from this study (LBN_CTKp3, LBN_CAKp91, and LBN_CTKp11) were extensively drug-resistant (XDR), exhibiting phenotypic resistance to all tested antibiotics except colistin; LBN_CAKp91 was additionally susceptible to tigecycline. Comparative analysis of antibiotic resistance genes and plasmid replicons revealed distinct resistome profiles among ST383 *K. pneumoniae* isolates (Fig. 1a).

The three study isolates shared an identical resistome with the previously reported Lebanese ST383 isolates, despite their phylogenetic divergence. The detailed resistome content of the three study isolates is presented in Supplemental material 3. This shared resistome comprised the ESBL genes *bla*_CTX-M-15_ and *bla*_CTX-M-14b_ alongside the carbapenemase genes *bla*_NDM-5_ and *bla*_OXA-48_, distributed across the same plasmid backbones: an IncL plasmid carrying *bla*_CTX-M-14b_ and *bla*_OXA-48_ and a hybrid IncHI1B(pNDM-MAR)/IncFIB(pNDM-MAR) plasmid carrying *bla*_CTX-M-15_ and *bla*_NDM-5_. These genes were conserved across all ST383 isolates analyzed, with two exceptions: ENG_KpvST383 lacked *bla*_OXA-48_, and EGY_K22 and EGY_DAPDKK01.1 harbored *bla*_NDM-1_ instead of *bla*_NDM-5_ (Fig. 1a).

The three study isolates harbored IncL and IncHI1B/FIB(pNDM-MAR). Additionally, ColRNAI plasmids were identified in LBN_CTKp3 and LBN_CAKp91, while LBN_CAKp91 also carried a Col440II plasmid. Among phylogenetically related strains, EGY_DAPDKA01.1 and ITA_CP141905 shared a similar resistome but differed in their plasmid content: Col440II plasmids were absent in EGY_DAPDKA01.1, and ColRNAI was detected only in this study isolates. In contrast, the hybrid IncHI1B(pNDM-MAR)/IncFIB(pNDM-MAR) plasmid, a hallmark of ST383, was present in all strains. The Egyptian isolates displayed more variable ESBL combinations: EGY_DAPDJW01.1 carried *bla*_CTX-M-19_ and *bla*_OXA-48_ on an IncL plasmid, while EGY_K22 harbored *bla*_CTX-M-14b_ and *bla*_OXA-48_ on IncL together with *bla*_NDM-1_. The co-occurrence of *bla*_NDM-5_, *bla*_OXA-48_, *bla*_CTX-M-15_, and *bla*_CTX-M-14b_ constitutes the predominant resistance profile among ST383 isolates in the EMR (Fig. 1a).

### Genetic environment of *bla*_NDM_

Two *bla*_NDM_ variants were identified across eight representative ST383 isolates from five countries: *bla*_NDM-1_ in EGY_DAPDKK01.1 and EGY_K22 and *bla*_NDM-5_ in the six remaining isolates (Fig. 1b).

In QAT_10MC160002271, ITA_CP141905, LBN_Kp1633, ENG_KpvST383, and the three isolates from the present study, *bla*_NDM-5_ is embedded within a conserved IS*15DIV-dsbD-ble-bla*_NDM-5_-IS*Spu2* segment and maintained in the same gene order across these complete assemblies. In our isolates, this segment was on a 369-kb IncHI1B/IncFIB(pNDM-MAR) hybrid plasmid. In EGY_DAPDKI01.1, contig fragmentation precluded assessment of the upstream IS*15DIV*-containing region; however, the *dsbD–ble–bla*_NDM_–IS*Spu2* core was identifiable, suggesting conservation of this cassette. EGY_DAPDKK01.1, harboring *bla*_NDM-1_, shared the conserved upstream region of the *bla*_NDM_ cassette.

Downstream of IS*630*, QAT_10MC160002271, ITA_CP141905, LBN_Kp1633, LBN_CTKp3, LBN_CTKp11, LBN_CAKp91, and ENG_KpvST383 carried an identical MDR region containing *aph(3’)-VI, qnrS1*, and *bla*_CTX-M-15_. Further downstream, *aac(6')-Ib, aadA1*, and *bla*_TEM-1_ were detected, associated with an integrase gene (Fig. 1b). EGY_DAPDKI01.1 showed the same downstream gene arrangement, although *aph(3')-VI* was not detected within the aligned segment of EGY_DAPDKI01.1.

EGY_K22 showed a clearly distinct organization. Upstream of *bla*_NDM-1_, only *dsbD*, *trpF*, and *ble* were present, with no IS*15DIV* or IS*630* detected.

### Genetic environment of *bla*_OXA-48_

Analysis of the genetic environment surrounding *bla*_OXA-48_ revealed a conserved arrangement among ST383 *K. pneumoniae* isolates (Fig. 1c). The gene is associated with a Tn*1999.2* composite transposon, characterized by IS*4*-like elements belonging to the IS*10A* family of transposases, flanking *bla*_OXA-48_ from the upstream and downstream. In addition, a *lysR* transcriptional regulator was found directly downstream of *bla*_OXA-48_, forming an IS*10A-bla*_OXA-48_-*lysR*-IS*10A* structure. The absence of flanking elements surrounding *bla*_OXA-48_ in EGY_DAPDKK01.1, EGY_DAPDKI01.1, and EGY_K22 is attributable to the draft nature of their genome assemblies.

### Comparative analysis of hypervirulence gene profiles

**Fig 2:**
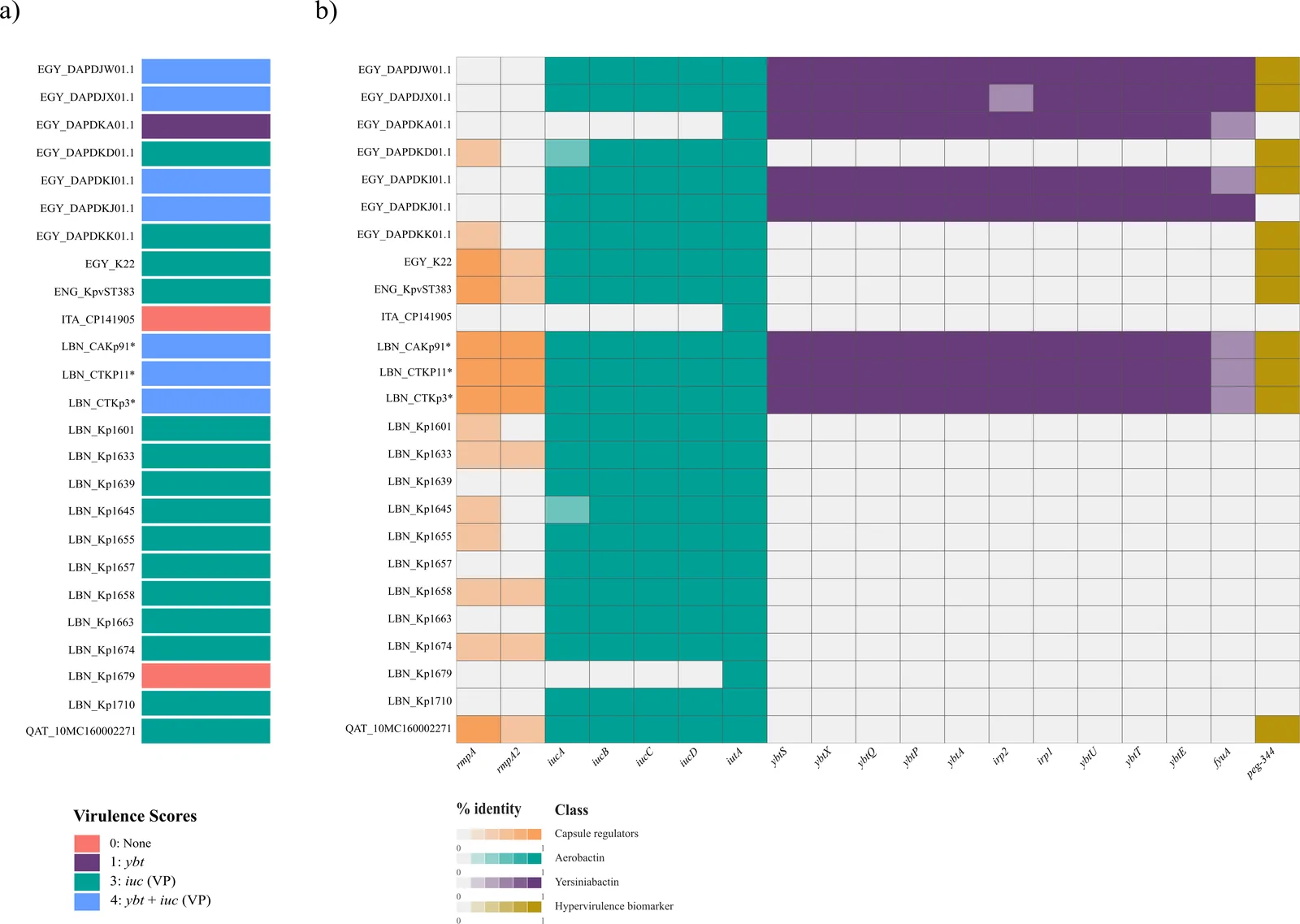
Hypervirulence gene profiles across ST383 *Klebsiella pneumoniae* isolates. (**a**) Virulence score profiles across ST383 *K. pneumoniae* isolates. Data shown summarize Kleborate results; isolates are shown as a single-column heatmap. Different colors indicate virulence score categories based on the presence of key virulence loci: 0 (none), 1 (yersiniabactin; *ybt*), 3 (aerobactin: *iuc*: VP: virulence plasmid), and 4 (*ybt + iuc*: VP). (**b**) Heatmap displaying the presence and percent nucleotide identity of hypervirulence-associated genes. Genes are grouped by functional class: capsule regulators (*rmpA* and *rmpA2*), aerobactin siderophore system (*iucA*, *iucB*, *iucC*, *iucD*, and *iutA*), yersiniabactin siderophore system (*ybtS, ybtX, ybtQ, ybtP, ybtA, irp2, irp1, ybtU, ybtT, ybtE,* and *fyuA*), and hypervirulence biomarker (*peg-344*). Color intensity represents percent nucleotide identity (0–1 scale), with full color indicating high identity and white indicating gene absence. Study isolates are marked with asterisks (*).

Comparative analysis of hypervirulence-associated genes revealed distinct virulence profiles among ST383 isolates (Fig. 2b). The three study isolates were the only strains to harbor the complete spectrum of hypervirulence determinants, including the capsule regulators *rmpA* and *rmpA2*, complete aerobactin siderophore (*iucABCD* and *iutA*) and yersiniabactin siderophore (*ybtAEPQSTUX*) loci, and the hypervirulence biomarker *peg-344*, all with high sequence identity. In contrast, all other ST383 isolates analyzed exhibited incomplete hypervirulence gene profiles. The Egyptian strain EGY_DAPDKI01.1 lacked *rmpA* and *rmpA2*. The Lebanese ST383 isolates reported by Sobh et al. (6) contained the aerobactin (*iuc*) locus in all isolates, except one. Low-identity matches to the capsule regulators *rmpA* and/or *rmpA2* were detected in a subset of isolates, whereas the yersiniabactin (*ybt*) locus and *peg-344* were absent from all genomes. The current study isolates represent the most hypervirulent sub-lineage of ST383 identified to date, with a virulence score of 4 (Fig. 2a), uniquely characterized by the convergence of all major virulence determinants.

### Structural organization of the virulence loci on the IncHI1B/IncFIB hybrid virulence plasmid

Due to identical genomic structures and minimal pairwise distances (three to nine single-nucleotide polymorphisms [SNPs]), LBN_CAKp91 was selected as the representative study isolate in the comparative alignment; all observations apply equally to LBN_CTKp3 and LBN_CTKp11.

**Fig 3:**
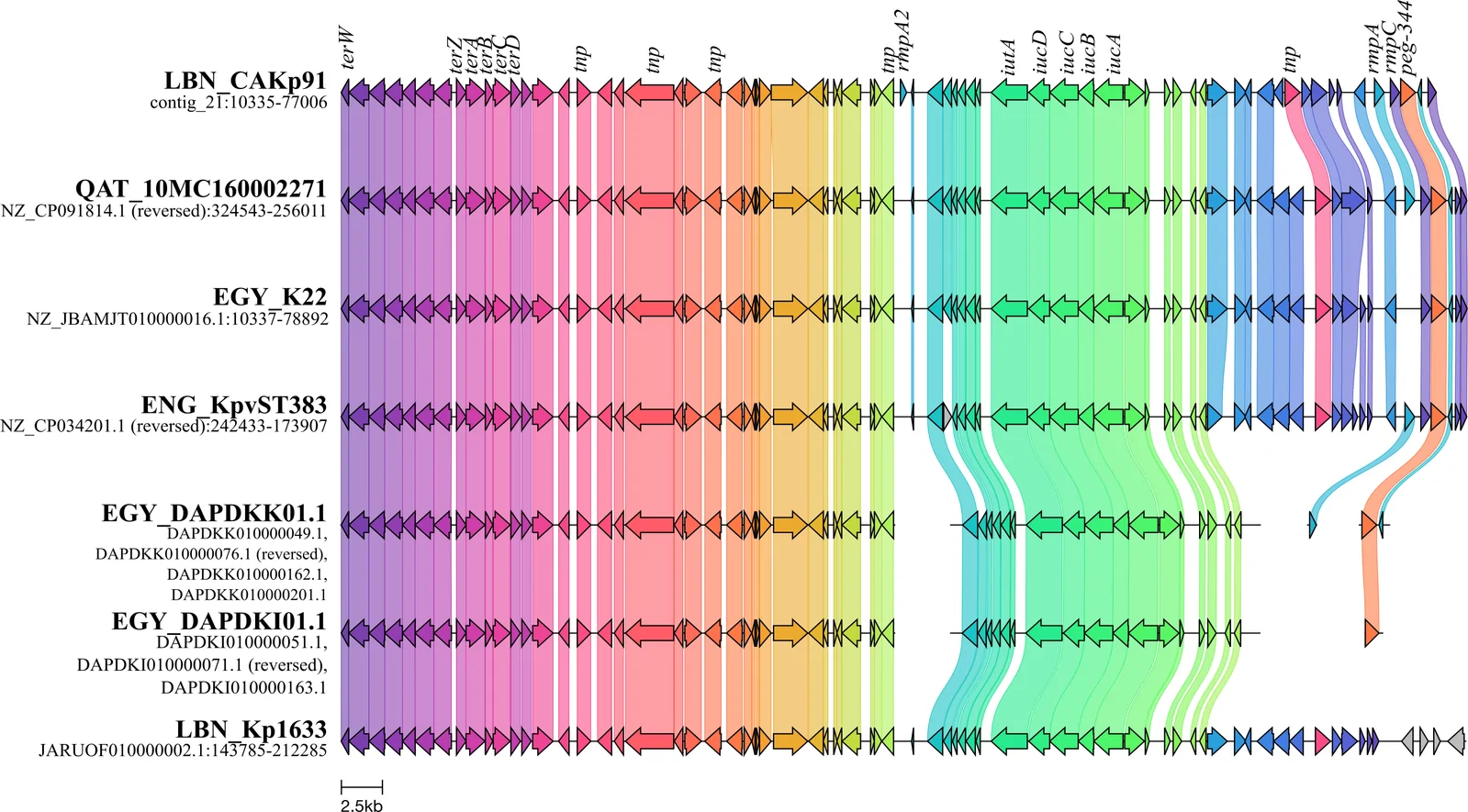
Structural organization of the virulence-associated plasmid region across representative ST383 *Klebsiella pneumoniae* isolates. Seven isolates spanning Lebanon (LBN), Egypt (EGY), England (ENG), and Qatar (QAT) are compared across a region encompassing the tellurite resistance operon (*terZABCDW*), aerobactin biosynthesis and uptake locus (*iucABCD*/*iutA*), and the hypermucoviscosity-associated cassette (*rmpA*, *rmpC*, *rmpA2*, and *peg-344*). Contig fragmentation in Egyptian draft assemblies reflects limitations of short-read sequencing and should be considered when interpreting apparent gene absence in the distal flanking regions. Asterisks denote study isolates.

The virulence region of the hybrid plasmid across seven representative ST383 isolates from Lebanon, Egypt, England, and Qatar showed a conserved central backbone comprising the aerobactin (*iucABCD-iutA*) and the tellurite resistance (*terZABCDW*) operons (Fig. 3).

Variation was restricted to the flanking *rmpA*, *rmpC*, and *peg-344* loci. LBN_CAKp91, ENG_KpvST383, and QAT_10MC160002271 retained all three genes, whereas EGY_K22 and EGY_DAPDKK01.1 lacked *rmpA and rmpC,* respectively. EGY_DAPDKI01.1 retained only peg-344, and LBN_Kp1633 lacked all three. Because the Egyptian genomes are draft assemblies, apparent absences in this region cannot be interpreted as confirmed gene losses. LBN_CAKp91 displayed the most complete configuration, carrying intact *rmpA* together with a truncated *rmpA2*.

### ICEKp comparative genomic structure analysis

**Fig 4:**
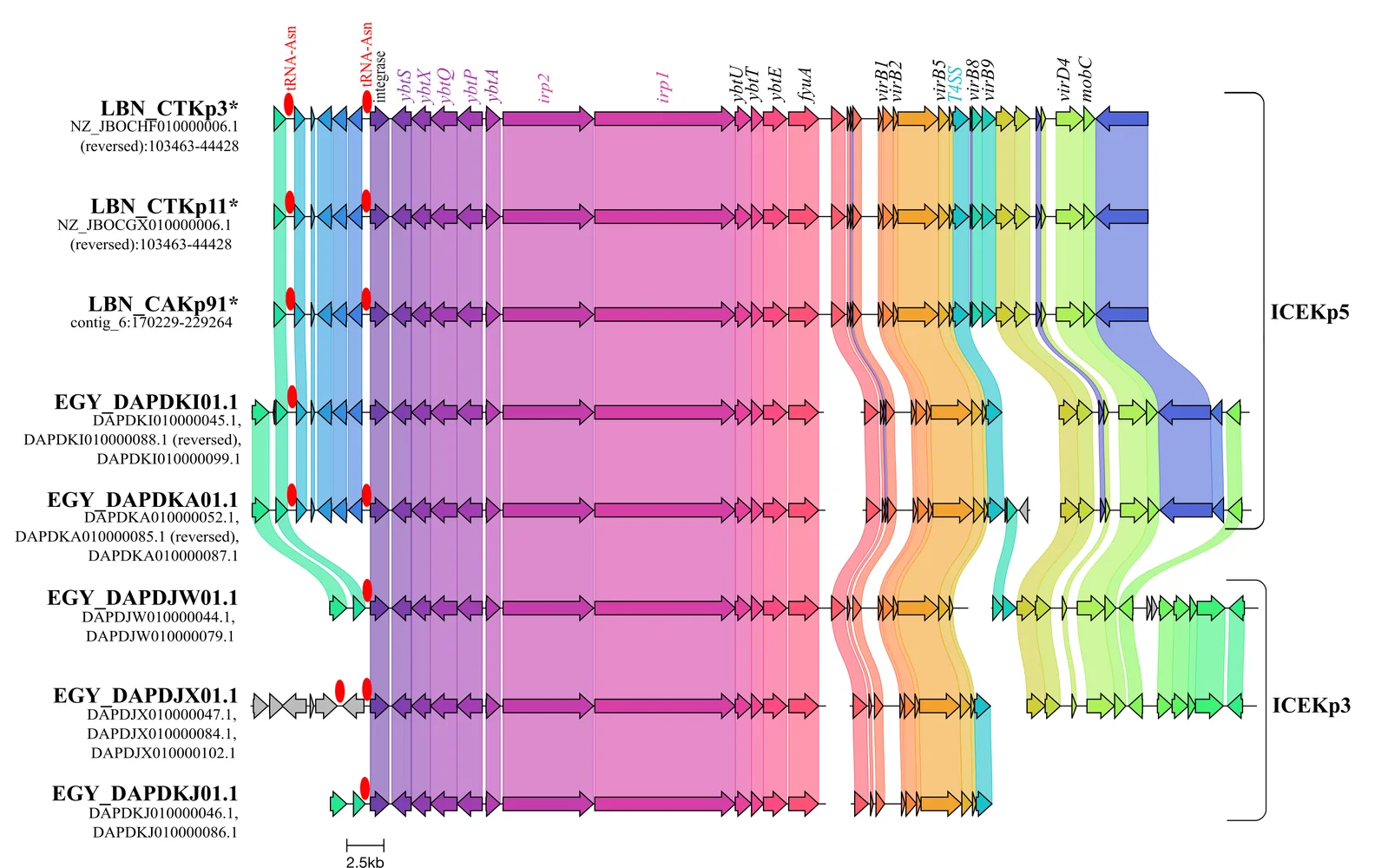
Linear comparison of ICEKp element structure across ST383 *Klebsiella pneumoniae* isolates. Gene blocks are represented as arrows indicating transcriptional direction and are colored by sequence identity, with colored ribbons connecting homologous regions between isolates. Red ovals denote the chromosomal tRNA-Asn integration sites characteristic of ICEKp elements. The conserved ICE architecture includes the following: an integrase gene at the left boundary; the complete yersiniabactin biosynthesis and transport locus (*ybtS*, *ybtX*, *ybtQ*, *ybtP*, and *ybtA*), *irp2* and *irp1*, and *ybtU*, *ybtT*, *ybtE*, *fyuA*; and the type IV secretion system (T4SS) conjugation machinery including *virB1*, *virB2*, and *virB5*, T4SS components, *virB8*, *virB9*, and *virD4*, and *mobC* at the right boundary. The scale bar represents 2.5 kb. Isolates are bracketed by ICEKp type as assigned by Kleborate: ICEKp5 (study isolates LBN_CTKp3*, LBN_CTKp11*, and LBN_CAKp91* and Egyptian isolates EGY_DAPDKI01.1 and EGY_DAPDKA01.1) and ICEKp3 (EGY_DAPDJX01.1, EGY_DAPDKJ01.1, and EGY_DAPDJW01.1). Asterisks (*) denote study isolates. Contig breaks in Egyptian draft assemblies reflect short-read fragmentation at repetitive ICE boundaries; synteny is preserved across assembled segments.

Comparative analysis of ICEKp elements demonstrated a conserved core organization across all eight ICE-positive ST383 isolates, independent of the geographic origin or ICEKp type (Fig. 4). All elements were integrated at the chromosomal tRNA-Asn site and shared the same structural framework: an integrase gene at the left boundary, the complete yersiniabactin biosynthesis and transport locus (*ybtS, ybtX, ybtQ, ybtP, ybtA, irp2, irp1, ybtU, ybtT, ybtE*, and *fyuA*), a type IV secretion system (T4SS) region at the right boundary (*virB1, virB2, virB5, virB8, virB9,* and *virD4*), and *mobC*. The yersiniabactin locus was intact in all isolates, with no internal disruptions in the recovered regions.

T4SS genes *virB1* and *virB2* were detected in all genomes, supporting retention of conjugative potential of the yersiniabactin locus. Contig breaks in the Egyptian draft assemblies were consistent with short-read fragmentation at repetitive ICE boundaries rather than true structural rearrangement as gene order remained conserved across aligned segments.

Despite this shared backbone, two distinct ICEKp configurations were observed, matching Kleborate assignments. The three study isolates, LBN_CTKp3, LBN_CTKp11, and LBN_CAKp91, carried ICEKp5 with the *ybt14* locus. Egyptian isolates, EGY_DAPDKI01.1 and EGY_DAPDKA01.1, also carried ICEKp5, whereas EGY_DAPDJW01.1, EGY_DAPDJX01.1, and EGY_DAPDKJ01.1 carried ICEKp3 with *ybt9*. The ICEKp3-positive isolates exhibited differences in flanking gene content compared with ICEKp5. EGY_DAPDJW01.1 and EGY_DAPDKJ01.1 were assigned YbST 183, while EGY_DAPDJX01.1 was assigned YbST 173-2LV due to two novel variants in *irp2* and *irp1* within the ICEKp3 backbone. All five ICEKp5-positive isolates shared YbST 151-1LV, a single variant of YbST 151, supporting a single ICEKp5 acquisition event from a common ancestor.

### SNP-based phylogenetic analysis

**Fig 5:**
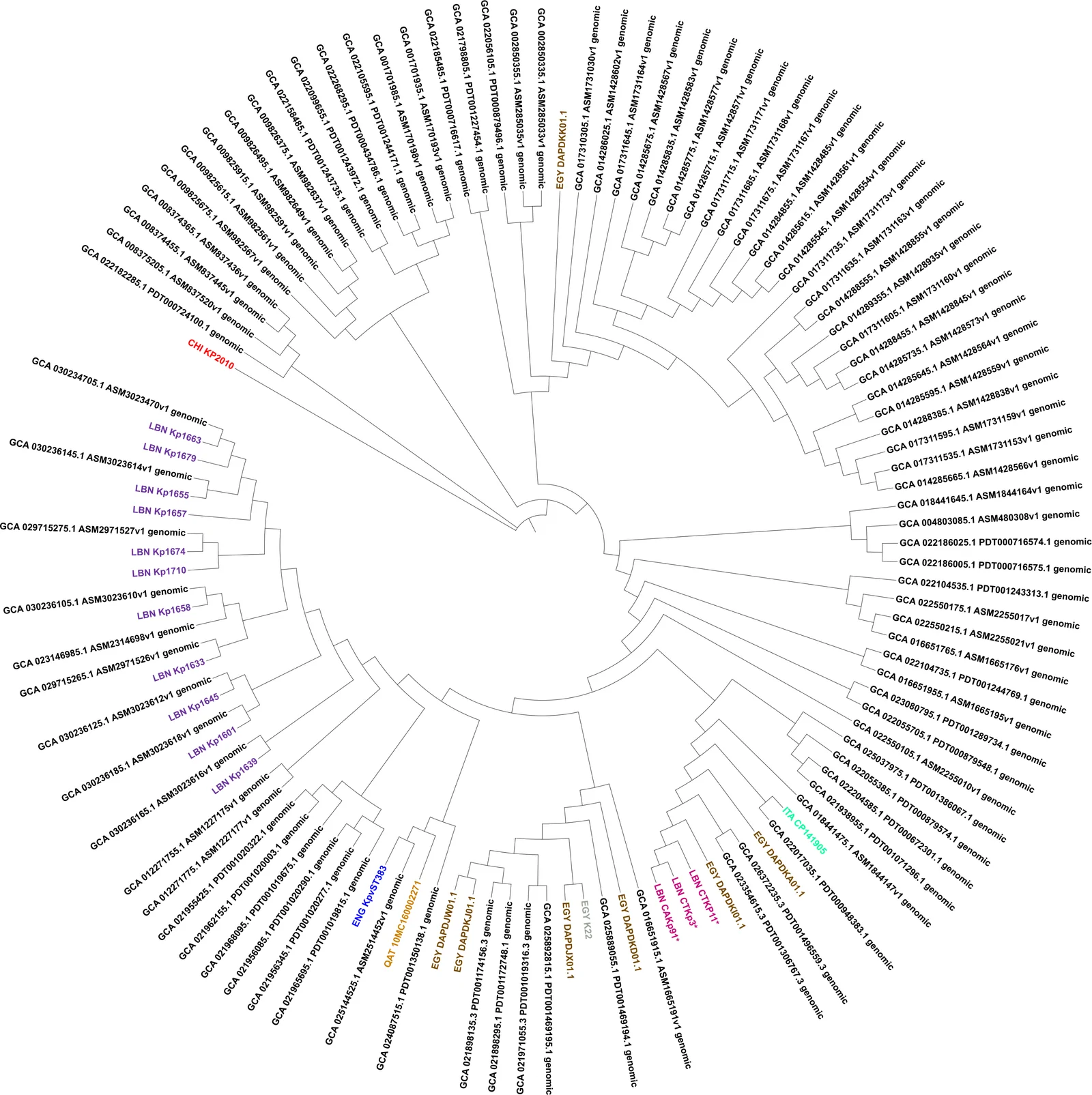
SNP-based phylogenetic analysis of ST383 *Klebsiella pneumoniae* isolates. Circular maximum-parsimony phylogenetic tree of 95 ST383 *K. pneumoniae* genomes retrieved from NCBI together with the three study isolates, rooted using CHI_KP2010 (red) as the outgroup. Tip labels are color-coded by geographic origin: study isolates LBN_CAKp91, LBN_CTKp3, and LBN_CTKp11 (pink); previously reported Lebanese ST383 isolates from reference 6 (purple); Egyptian isolates (orange); Qatari isolate QAT_10MC160002271 (teal); English isolate ENG_KpvST383 (blue); Italian isolate ITA_CP141905 (green); outgroup CHI_KP2010 (red). Unlabeled tips represent additional publicly available ST383 assemblies retrieved from NCBI. The three study isolates form a well-supported monophyletic cluster, most closely related to the Egyptian strain EGY_DAPDKI01.1 and clearly distinct from the previously reported Lebanese ST383 isolates (>164 pairwise SNPs). Core SNPs were called using Snippy v4.6 with QAT_10MC160002271 as the reference genome; the parsimony tree was constructed using kSNP3 v3.0 and visualized using iTOL.

SNP-based phylogenetic reconstruction of ST383 *K. pneumoniae* isolates revealed distinct phylogenetic clustering associated with geographic origins (Fig. 5). The study isolates (LBN_CAKp91, LBN_CTKp3, and LBN_CTKp11) formed a well-supported monophyletic clade, separated by only three to nine pairwise SNPs, and grouped most closely with EGY_DAPDKI01.1. LBN_CTKp3 exhibited only 59 SNP differences from EGY_DAPDKI01.1, while LBN_CAKp91 and LBN_CTKp11 showed 60 and 65 SNPs, respectively, from this Egyptian strain (Supplemental material 2).

The next closest were the isolates from Qatar (108–114 SNPs), Italy (120–126 SNPs), and another from Egypt (150–156 SNPs). In contrast, a substantial divergence was observed from the Sobh et al. (6) Lebanese isolates (range: 164–220 SNPs) (Fig. 5 and Supplemental material 2). The EGY_K22 isolate, previously reported to have similar virulence determinants including the aerobactin locus and IncHI1B/IncFIB virulence plasmid, showed SNP distances of 175–181 from the study isolates. These data point to a distinct ST383 introduction into Lebanon, more closely related to Egyptian strains than to previously reported local lineages.

### Convergent evolution of ST383 in Lebanon toward an hv-XDR phenotype

The three study isolates, CTKp3, CTKp11, and CAKp91, represent an emerging, phylogenetically distinct ST383 sub-lineage converging hypervirulence and extensive drug resistance through stepwise acquisition of mobile genetic elements. ST383, an independent non-CG258 lineage, has progressed over the past decade from an OXA-48-producing ESBL clone first described in the United Kingdom in 2019 to a globally disseminated high-risk clone across the EMR. These isolates carry an ICEKp5 (~75–80 kb) integrated at the tRNA-Asn site, encoding the yersiniabactin locus *ybt14* and a functional T4SS, alongside an IncL pOXA-48 plasmid and an IncFIB/IncHI1B hybrid plasmid encoding *bla*_NDM-5_, *bla*_CTX-M-15_, the aerobactin operon (*iucABCD* and *iutA*), and hypermucoidy regulators *rmpA* and *rmpA2*. This genomic configuration yields a Kleborate virulence score of 4, classifying these isolates as convergent hypervirulent.

## DISCUSSION

*K. pneumoniae* ST383 has emerged as a high-risk lineage increasingly associated with the convergence of MDR and hypervirulence across the EMR and beyond (6, 11, 13, 29). In Lebanon, ST383 was previously recognized as a carbapenem-resistant hospital-adapted clone carrying *bla*_NDM-5_ and *bla*_OXA-48_ on IncFIB-IncHI1B and IncL plasmids, respectively (6). In that cohort, 26 of 34 *K. pneumoniae* isolates recovered between 2019 and 2021 belonged to ST383 and differed by only 4–80 SNPs, consistent with local clonal expansion. However, it lacked evidence of hypervirulence-associated elements such as ICEKp5. This study reports the detection of a phylogenetically distinct ST383 *K. pneumoniae* sub-lineage in Lebanon, defined by the acquisition of ICEKp5, which carries the yersiniabactin virulence locus, and harbors the full complement of hypervirulence determinants and dual carbapenemase genes. These findings highlight the ongoing convergence of hypervirulence and XDR within this high-risk clone.

The three *K. pneumoniae* ST383 isolates characterized in this study are distinct (164–220 SNPs) from the previous Lebanese ST383 isolates (6), indicating an independent introduction of ST383 into Lebanon. The study isolates clustered closely with Egyptian ST383 strains, particularly EGY_DAPDKI01.1. LBN_CTKp3, LBN_CAKp91, and LBN_CTKp11 exhibited 59, 60, and 65 SNPs, respectively, from this Egyptian strain. The next closest were the isolates from Qatar (108–114), Italy (120–126), and another from Egypt (150–156). This phylogenetic proximity to Egyptian and regional strains suggests a potential epidemiological link, possibly through healthcare-associated travel, patient transfer, or shared transmission networks within the EMR.

A key feature distinguishing the present isolates from those reported by Sobh et al. (6) is the presence of ICEKp5 carrying the yersiniabactin locus *ybt14* (*ybt* ST151). This element, integrated at the chromosomal tRNA-Asn site, contains the complete yersiniabactin biosynthesis and transport locus and a functional T4SS enabling conjugative transfer. In addition, the study isolates harbor a convergent IncHI1B/IncFIB plasmid encoding the full aerobactin locus (*iucABCD* and *iutA*) together with hypermucoidy regulators (*rmpA, rmpA2, rmpC,* and *rmpD*). This combination results in a Kleborate virulence score of 4, classifying them as convergent XDR hypervirulent strains. The colocalization of the tellurite resistance operon *terZABCDW* with the aerobactin locus and hypermucoidy regulators on the same plasmid backbone suggests that tellurite exposure in hospital environments may represent an additional, antibiotic-independent selective pressure favoring plasmid maintenance and contributing to the nosocomial persistence of this convergent clone. The absence of the colibactin (*clb*) and salmochelin (*iro*) loci further defines a specific virulence gene repertoire characteristic of this ST383 sub-lineage (13).

Among Egyptian isolates, EGY_DAPDKI01.1 and EGY_DAPDKA01.1 carried ICEKp5, whereas EGY_DAPDJW01.1, EGY_DAPDJX01.1, and EGY_DAPDKJ01.1 carried ICEKp3 with *ybt9* (11). All five ICEKp5-positive isolates shared YbST 151-1LV, a single variant of YbST 151, supporting a single ICEKp5 acquisition event from a common ancestor. The conserved ICEKp5 configuration across the three Lebanese study isolates and two Egyptian strains supports close phylogenetic relatedness and defines a distinct sub-lineage separate from the ICEKp3-dominant Egyptian ST383 isolates that have emerged within the EMR. The yersiniabactin siderophore system is important for iron acquisition in nutrient-limited host environments such as the urinary tract and bloodstream (30). Yersiniabactin has been established as a virulence factor for *K. pneumoniae* during pulmonary infection, with yersiniabactin mutants showing attenuated virulence in murine models (30). The acquisition of ICEKp5 by the study isolates represents a significant step toward convergent hypervirulence. This comparative analysis of hypervirulence-associated genes further showed that the study isolates were the only ST383 strains to harbor the complete spectrum of hypervirulence determinants, including *rmpA* and *rmpA2*, the aerobactin siderophore system (*iucABCD* and *iutA*) on the IncHI1B/IncFIB hybrid virulence plasmid, yersiniabactin on ICEKp5, and the hypervirulence biomarker *peg-344*, which is absent from the Sobh et al. (6) Lebanese ST383 cohort.

ST383 demonstrates a progressive evolutionary trajectory driven by repeated acquisition and reshuffling of major carbapenemase and virulence determinants. Despite their phylogenetic distinctiveness and divergent virulence profiles, they share an identical resistance gene profile. Globally disseminated ST383 strains from Egypt, Lebanon, Qatar, and Italy (13, 29, 31) co-harbored *bla*_OXA-48_ together with either *bla*_NDM-1_ or *bla*_NDM-5_, ESBLs such as *bla*_CTX-M-15_ and *bla*_CTX-M-14_, indicating the incorporation of different NDM variants during the acquisition of the large IncHI1B/IncFIB hybrid resistance and virulence plasmid. Analysis of the genetic environment surrounding *bla*_OXA-48_ revealed a highly conserved structure across all isolates, suggesting a common origin of the *bla*_OXA-48_ -carrying IncL(pOXA-48) plasmid and its stable maintenance within the ST383 lineage. Similarly, the *bla*_NDM_ genetic environment was highly conserved, with most isolates carrying *bla*_NDM-5_. The conserved structure included an MDR module conferring resistance to carbapenems, aminoglycosides, quinolones, and other β-lactams, severely limiting therapeutic options.

In Lebanon and Egypt, sequenced ST383 isolates predominantly carried *bla*_NDM-5_, whereas *bla*_NDM-1_ on related hybrid plasmids was identified in other lineages such as ST11, demonstrating lineage-specific variation and accessory genome plasticity. Earlier European ST383 isolates carried alternative carbapenemases, including *bla*_VIM-4_, *bla*_VIM-19_, and *bla*_KPC-2_, revealing that this clone has repeatedly replaced resistance determinants over time (32). Phylogenetic and plasmid data support a model of sequential acquisition, with ancestral ST383 first acquiring an IncL plasmid bearing *bla*_OXA-48_, followed by integration of a large IncHI1B fusion plasmid carrying *bla*_NDM_ variants and virulence loci, transforming a conventional MDR lineage into a convergent, highly adaptable pathogen. Our findings indicate that ST383 in Lebanon is not solely persisting through local hospital expansion but embraces a distinct introduced lineage. The detection of ICEKp5, absent from previously reported Lebanese ST383 genomes, along with clear phylogenetic separation, supports an independent introduction event rather than local microevolution of the earlier circulating clone. These findings emphasize that ST383 continues to evolve through regional dissemination and stepwise genetic acquisition, highlighting its growing impact on clinical management and infection control across interconnected healthcare systems.

This study documents the identification of a phylogenetically distinct ST383 *K. pneumoniae* sub-lineage in Lebanon, characterized by convergence of XDR and hypervirulence following acquisition of ICEKp5. The three study isolates uniquely harbor all hypervirulence determinants, including *rmpA, rmpA2*, aerobactin, yersiniabactin, and *peg-344*. By also co-harboring *bla*_OXA-48_ and *bla*_NDM-5_, they represent the most extensively characterized hv-XDR ST383 variant reported to date. Phylogenetic analysis confirmed the independent introduction of these isolates, distinct from the previously endemic Lebanese clone and closely related to strains circulating in the region. These findings highlight regional dissemination, emphasizing the need for enhanced genomic surveillance, strengthened infection-control measures, and coordinated regional efforts to limit the spread of this emerging high-risk clone in the EMR.

This study has several limitations, including the absence of *in vivo* validation of the hypervirulence phenotype and the lack of epidemiological data, which prevented definitive identification of the source of introduction.

## Data Availability

The draft genomes and hybrid assemblies have been deposited at the National Center for Biotechnology Information (NCBI) database. The accession numbers for these isolates are listed in Supplemental material 1.

## SUPPLEMENTAL MATERIAL

Supplemental material 1 aac.00384-26-s0001.xlsx Accession numbers for the study isolates and all genomes retrieved from NCBI.

Supplemental material 2 aac.00384-26-s0002.tiff Heatmap displaying pairwise core-genome SNP distances among the ten ST383 **K. pneumoniae** isolates showing the closest phylogenetic relatedness to the three study isolates, as determined from the 95-genome SNP-based phylogenetic analysis.

Supplemental material 3 aac.00384-26-s0003.xlsx Complete resistome profile for the study isolates.

ASM does not own the copyrights to Supplemental Material that may be linked to, or accessed through, an article. The authors have granted ASM a non-exclusive, world-wide license to publish the Supplemental Material files. Please contact the corresponding author directly for reuse.
